# Critical behavior near the reversible-irreversible transition in periodically driven vortices under random local shear

**DOI:** 10.1038/s41598-019-51060-9

**Published:** 2019-11-11

**Authors:** S. Maegochi, K. Ienaga, S. Kaneko, S. Okuma

**Affiliations:** Department of Physics, Tokyo Institute of Technology, 2-12-1, Ohokayama, Meguro-ku, Tokyo 152-8551 Japan

**Keywords:** Nanoscience and technology, Physics

## Abstract

When many-particle (vortex) assemblies with disordered distribution are subjected to a periodic shear with a small amplitude ${\boldsymbol{d}}$, the particles gradually self-organize to avoid next collisions and transform into an organized configuration. We can detect it from the time-dependent voltage ${\boldsymbol{V}}{\boldsymbol{(}}{\boldsymbol{t}}{\boldsymbol{)}}$ (average velocity) that increases towards a steady-state value. For small ${\boldsymbol{d}}$, the particles settle into a reversible state where all the particles return to their initial position after each shear cycle, while they reach an irreversible state for ${\boldsymbol{d}}$ above a threshold ${{\boldsymbol{d}}}_{{\boldsymbol{c}}}$. Here, we investigate the general phenomenon of a reversible-irreversible transition (RIT) using periodically driven vortices in a strip-shaped amorphous film with random pinning that causes local shear, as a function of ${\boldsymbol{d}}$. By measuring ${\boldsymbol{V}}{\boldsymbol{(}}{\boldsymbol{t}}{\boldsymbol{)}}$, we observe a critical behavior of RIT, not only on the irreversible side, but also on the reversible side of the transition, which is the first under random local shear. The relaxation time ${\boldsymbol{\tau }}{\boldsymbol{(}}{\boldsymbol{d}}{\boldsymbol{)}}$ to reach either the reversible or irreversible state shows a power-law divergence at ${{\boldsymbol{d}}}_{{\boldsymbol{c}}}$. The critical exponent is determined with higher accuracy and is, within errors, in agreement with the value expected for an absorbing phase transition in the two-dimensional directed-percolation universality class. As ${\boldsymbol{d}}$ is decreased down to the intervortex spacing in the reversible regime, ${\boldsymbol{\tau }}{\boldsymbol{(}}{\boldsymbol{d}}{\boldsymbol{)}}$ deviates downward from the power-law relation, reflecting the suppression of intervortex collisions. We also suggest the possibility of a narrow smectic-flow regime, which is predicted to intervene between fully reversible and irreversible flow.

## Introduction

Many-particle systems subjected to external driving forces exhibit a variety of nonequilibrium phases and phase transitions, such as a plastic depinning transition^1–11^ and a reversible-to-irreversible flow transition (RIT)^12–17^. Using a superconducting vortex system in amorphous ($a$-) Mo${}_{x}$Ge${}_{1-x}$ films with random pinning centers, we have previously shown^18–20^ that the depinning transition is a nonequilibrium phase transition, as predicted numerically^5^: When the vortices (particles) with an ordered initial configuration are driven by a suddenly applied dc current (dc force), they are gradually pinned by random pinning sites and transform into a less organized configuration. This transient process called a dynamic disordering is detected from the time-dependent voltage $V(t)$, corresponding to the average velocity of vortices, induced by vortex motion that decays toward a steady-state voltage ${V}^{\infty }(\equiv V(t\to \infty ))$. Relaxation times $\tau$ required for the system to settle into the steady state exhibit a power-law divergence at the depinning current with critical exponents ${\nu }_{d}=1.4\pm 0.4$^18–20^. More recently, using ac drive^21^, we have also observed the critical behavior of the depinning transition with the critical exponent close to that for the dc drive, which is the further demonstration of the universality of the nonequilibrium depinning transition^5^. In these experiments, however, the critical behavior has been observed only on the moving (fluctuating diffusing [active]) side of the transition. This is because a reliable data of $V(t)$ could not be obtained in the pinned (non-fluctuating quiescent [absorbing]) phase, where ${V}^{\infty }$ = 0. The diverging $\tau$ on both sides of the transition has been reported in the single crystal of NbS${}_{2}$^22^. Note, however, that the transition is induced by “jamming" of vortices at large dc currents instead of the depinning at small currents. Reported values of $\tau$ are by approximately five orders of magnitude larger than those in the depinning transition and the critical exponent $(=1.6\pm 0.12)$ is somewhat larger than ${\nu }_{d}\,=$ 1.4, suggestive of the different universality class of the “jamming" transition from that of the depinning transition.

In observing the depinning transition, the random pinning centers within the amorphous films play a crucial role. Meanwhile, independent of pinning, when the particles (vortices) with a random initial distribution are periodically driven by a suddenly applied ac shearing force (ac current), they collide with one another and experience a random force^12–15^. This gives rise to a rearrangement in the particle configuration so that they avoid next collisions, thus random organization (the dynamic ordering) proceeds. After the long-time periodic shear with small displacements, the particles settle into a reversible state where all of them return to their initial position after each shear cycle and hence, it is a non-fluctuating quiescent (absorbing) state. On the other hand, they reach a fluctuating irreversible (active) state for large shear amplitudes $d$ above a threshold value ${d}_{c}$, where some particles always collide and the system loses reversibility. The relaxation times $\tau$ for the system to reach the steady state show a power-law divergence on both sides of the transition, indicative of the nonequilibrium phase transition. This phenomenon called RIT was first observed in the experiment of colloidal suspensions which were contained and sheared periodically in the gap between two concentric cylinders and in the numerical simulation^12–14^. The critical exponents obtained from $\tau (d)$ for the experiment and for the simulation in two dimensions (2D) were $\nu \,=\,$1.1 $\pm$ 0.3 and 1.33 $\pm$ 0.02, respectively. These values are similar to ${\nu }_{d}$ for the depinning transition^18–20^, suggesting that the both transitions may fall into the same universality class^5^.

To explore the universality of RIT reported in the colloidal suspensions, we performed similar experiments using the vortex system of the $$a$$-Mo$${}_{x}$$Ge$${}_{1-x}$$ films. First, we used a film with a Corbino-disk (CD) contact geometry^18^. In CD, under the application of an ac radial current, the vortices rotate periodically back and forth around the CD center by feeling a global shear inversely proportional to the radius $$r$$ of rotation, where they do not cross the sample edges^23–25^. Although this is analogous to the colloidal experiment^12–14^, there is some difference between the two systems: The colloidal system^12–14,17^ is a 3D and dilute system, while the vortex system is a 2D and more strongly interacting system. For the latter system in the $$a$$-Mo$${}_{x}$$Ge$${}_{1-x}$$ film, the characteristic length scales for the vortex core and vortex-vortex interaction are the superconducting coherence length and London penetration length, respectively, which are of the order of $$\approx $$ 1 $$\times $$ 10 and $$\approx $$$$1{0}^{2}$$ nm^26,27^. In contrast to the case of the colloidal system, the collisions between the vortices do not occur directly. One can easily control the mean intervortex spacing $${a}_{0}\approx \sqrt{{\Phi }_{0}/B}$$ by changing the applied magnetic field $$B$$, which ($${a}_{0}\,\approx $$ 3 $$\times $$ 10 nm) is of the order of the core size for $$B\, \sim $$ 3.5 T used in this study, where $${\Phi }_{0}$$ is a flux quantum. Despite these differences between the two systems, we observed the critical behavior of RIT in the vortex system^15^ similar to that reported in the colloidal suspensions.

The study of the vortex system has implications, since the results are compared with the ones from more strongly interacting systems, such as dense, amorphous solid^28–30^ and jamming systems^31–33^, and from more dilute colloidal ones^12–14,17^, which undergo RIT. In more recent simulations, the critical behavior of RIT with similar $\nu =1.2\mbox{--}1.3$ has been reported for the colloidal suspensions subjected to isotropic local shear^34^, instead of the anisotropic global shear mentioned above. It has been predicted that in the vicinity of RIT, large-scale density fluctuations in the particle configuration are suppressed and the particular configuration called hyperuniform order emerges. Recently, we extended our study of RIT using CD with the artificial global shear ($\propto 1/r$) to cover more general situations where the random local shear due to random quenched disorder is present. Thus, we used ordinary strip-shaped films of $a$-Mo${}_{x}$Ge${}_{1-x}$ with random pinning centers and found that RIT also occurs^35,36^, consistent with the theoretical prediction^15^.

In our previous study using a vortex system^18,35,36^, however, the experimental resolution of the time-dependent voltage $$| V(t)| $$ was not sufficient to resolve the critical dynamics accurately, in particular, in the reversible state. In fact, we could not even detect the critical behavior of RIT in the reversible state of the strip samples^35,36^. The critical exponent $$\nu $$ obtained from the CD^18^ and strip samples^35,36^ had large errors, e.g., $$\nu =1.3\pm 0.3$$ and $$1.35\pm 0.15$$, respectively, making it difficult to characterize the details of the transition, such as the universality class of the transition, in a convincing manner. We consider that the similar difficulties will be experienced by other experimental systems studying RIT^12–14,30^. To overcome the difficulties, in our work, we have used the ac current with much higher frequencies $$f$$ than before, thus significantly improving the time resolutions of the relaxation curve $$| V(t)| $$ toward the steady state as well as the resolutions of $$d$$. As a result, we have clearly observed the critical relaxation $$\tau (d)$$ not only on the irreversible side but also on the reversible side of the transition, and obtained the critical exponent $$\nu =1.38\pm 0.08$$ with reduced error bars. The thus obtained $$\nu $$ is, within error bars, in agreement with the critical exponent $$\nu =1.295\pm 0.006$$ expected for the absorbing phase transition^37–41^ in the directed-percolation (DP) universality class in 2D^42,43^.

We have also succeeded in obtaining detailed information on the reversible flow and the transition region. We have found that as $d$ is decreased down to the average intervortex spacing ${a}_{0}$ in the reversible state, the $\tau (d)$ shows an abrupt downward deviation from the power-law relation and takes very small values at $d\lesssim {a}_{0}$. We interpret this behavior as reflecting the suppression of vortex-vortex collisions and random organization at $d\lesssim {a}_{0}$. Finally, we will present a heuristic discussion on the possibility of a smectic-flow regime, which has been predicted^44^ to intervene between reversible and irreversible flow.

## Experimental Method

The $$a$$-Mo$${}_{x}$$Ge$${}_{1-x}$$ film with thickness of 0.33 $$\mu $$m was fabricated by radio-frequency sputtering deposition onto a Si substrate mounted on a water cooled copper stage that rotates at 240 rpm^18,20,25,35,36^. The superconducting transition temperature $${T}_{c}$$ at which the resistivity $$\rho $$ falls to zero is 6.3 K in zero magnetic field ($$B=0$$). The field $$B$$ was applied perpendicular to the film surface. By applying a current, the vortices induced by $$B$$ move in the direction parallel to the film width of 0.3 mm. The voltage $$V$$ induced by vortex motion was measured by using voltage probes separated at $$l\,=$$ 1.2 mm. We measured $$\rho $$ in the linear regime and $$V$$ with a standard four-probe method. The time-evolution of the voltage $$V(t)$$ immediately after applying the ac current $${I}_{ac}$$ of square waveform was measured^18,25,35^ by using an oscilloscope (Rohde and Schwarz RTO2024) with 10 MHz. To improve significantly the time resolution of $$V(t)$$ and obtain the reliable relaxation curve of the voltage amplitude $$| V(t)| $$, the frequency $$f$$ of the ac current $${I}_{ac}$$ was fixed to be a high value of 450 kHz, which is more than an order of magnitude higher than $$f\,=$$ 0.6–20 kHz used in our previous study^18,35,36^. The amplitude of $${I}_{ac}$$ was adjusted to generate the steady-state voltage $${V}^{\infty }(\equiv | V(t\to \infty )| )$$ with desired values. The film was directly immersed into the liquid $${}^{4}$$He.

## Results and Discussion

For a weak pinning superconductor, such as the $a$-Mo${}_{x}$Ge${}_{1-x}$ film studied here, a vortex-solid state is a weakly disordered vortex lattice or Bragg glass. In the high field region below the upper critical field, the depinning current ${J}_{d}$, at which the dc voltage appears with increasing the dc current, exhibits a peak as a function of the field $B$ or temperature $T$, which originates from softening of the vortex lattice just prior to melting and from random pinning due to quenched disorder^25^. This is called a peak effect and all measurements in this work were performed in the so-called peak-effect regime^45–49^ at 3.5 T and 4.1 K, where the pinning is very effective^18,25^. The average intervortex spacing ${a}_{0}$ is evaluated to be 26 nm from the field value of 3.5 T. To achieve random organization associated with transient vortex dynamics near RIT, we need to prepare initial vortex assemblies with a disordered configuration in which many vortices are pinned by random pinning sites. For this purpose, we have driven the vortices using a small ac current of 12 kHz yielding ${V}^{\infty }\,=$ 100 $\mu$V for a long time, more than 4 $\times 1{0}^{3}$ cycles, to reach the steady state. From the dc current-voltage characteristics, which show an upward curvature below about 1 mV, we know that 100 $\mu$V corresponds to a disordered plastic-flow state dominated by pinning^18–20,25,36^. Using a simple relation $d={V}^{\infty }/(2lfB)$, the displacement amplitude $d$ for the periodically sheared vortices in the steady state ($t\to \infty$) is estimated to be around 1 $\mu$m, which is much larger than the critical displacement ${d}_{c}(\approx 45\ {\rm{nm}})$ of RIT, as described below. Therefore, the frozen vortex configuration obtained by abruptly switching off the driving current is highly disordered^20,36,50^.

**Figure 1:**
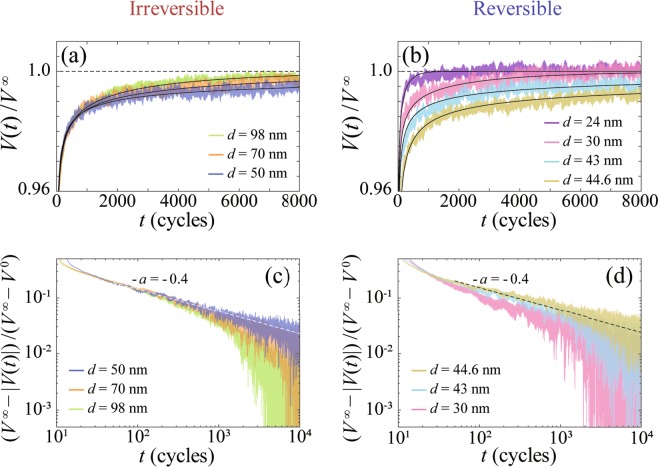
Random organization associated with transient vortex dynamics near RIT. (**a**,**b**) Voltage responses $$V(t)/{V}^{\infty }$$ for the disordered initial vortex configuration subjected to the ac drive with various shear amplitudes: (**a**) $$d\,=$$ 98.4, 69.9, and 49.9 nm, and (**b**) $$d\,=$$ 24.0, 30.0, 42.6, and 44.6 nm from top to bottom. Here, the voltage $$V(t)$$ is normalized by the steady-state voltage $${V}^{\infty }$$. For clarity, vertical lines of the individual voltage pulses are removed from the graphs and only the amplitude of the pulse, $$| V(t)| $$, is shown. Full lines in (**a**,**b**) indicate the fits of $$| V(t)| /{V}^{\infty }$$ to Eq. (1) and horizontal dashed lines represent $$| V(t)| /{V}^{\infty }$$ = 1. (**c**,**d**) Replots of the data shown in (**a**,**b**), as $$({V}^{\infty }-| V(t)| )/({V}^{\infty }-{V}^{0})$$ versus $$t$$, on a log-log scale: (**c**) $$d\,=$$ 49.9, 69.9, and 98.4 nm, and (**d**) $$d\,=$$ 44.6, 42.6, and 30.0 nm from top to bottom. Dashed lines in (**c**,**d**) indicate a slope of $$-a=-\,0.40(\pm 0.05)$$, nearly consistent with the theoretical value of $$a\approx 0.45$$ for the DP universality class in 2D^42,51^.

The thus prepared disordered initial vortex configuration is then subjected to the ac current $${I}_{ac}$$ of fixed $$f=$$ 450 kHz with various amplitudes $$| {I}_{ac}| $$. Here, the displacement $$d$$ for the ac motion in the steady state was varied in the range $$d\,=$$ 12–100 nm, which corresponds to $$d\simeq 0.5{a}_{0}$$–4$${a}_{0}$$, by changing $${V}^{\infty }$$ from 45 to 370 $$\mu $$V.Representatively shown in Fig. 1(a,b) are the voltage responses $$V(t)/{V}^{\infty }$$ of the system to the ac drive with $$d\,=$$98.4, 69.9, and 49.9 nm from top to bottom and $$d\,=$$44.6, 42.6, 30.0, and 24.0 nm from bottom to top, respectively, where the voltage $$V(t)$$ is normalized by $${V}^{\infty }$$. To clearly see the difference in the relaxation time between the several graphs in each figure, the large voltage region, $$| V(t)| /{V}^{\infty }\, > $$ 0.96, is enlarged and shown. For clarity, vertical lines of the individual voltage pulses are removed from the graphs and only the amplitude of the pulse, $$| V(t)| $$, is shown.

For all the data, the normalized amplitude of the voltage, $| V(t)| /{V}^{\infty }$, exhibits a monotonic increase with $t$ and a relaxation toward a steady-state value of unity. Since the voltage is proportional to the average velocity of the vortices in the system, the results indicate that an initial vortex flow at $t \sim 0$ is a disordered flow dominated by pinning, where the vortices cannot move easily. However, being periodically driven over the random pinning potential, the vortices collide with one another, facilitating a rearrangement in the distribution to avoid future collisions. Finally, the system arrives at a less disordered state in which the vortices are easier to move than in the initial state^36,50^. The similar behavior was observed in the colloidal suspensions^14^. The data of Fig. 1(a) indicates that the relaxation is longer for smaller $d$, while that in Fig. 1(b) shows the longer relaxation for *larger* $d$, indicating that a peak in the relaxation time $\tau (d)$ occurs at around $d\,=$ 45–50 nm.

To extract $$\tau $$ for the system to reach the steady state, we fit the amplitude of the transient voltage, $$| V(t)| $$, using the following relaxation function presented in^5,14^:

1 $$| V(t)| ={V}^{\infty }-({V}^{\infty }-{V}^{0})exp(-t/\tau )/{t}^{a}.$$

Here, ${V}^{0}$ and ${V}^{\infty }$ are the initial and steady-state voltage amplitudes, and $\tau$ is the characteristic time at which the relaxation crosses over from a power-law decay with an exponent $a$, as shown later in Fig. 1(c,d), to an exponential decay. Hence, $a$ is relevant very close to the transition or at small $t$ such that $\tau /t\,\gg$ 1^5,11,14,44^. The best fit is obtained with $a\,=$ 0.40 $\pm \,0.05$. In Fig. 1(c,d), we replot all the data (except for the data of $d\,=$ 24 nm) shown in Fig. 1(a,b), respectively, as $({V}^{\infty }-| V(t)| )/({V}^{\infty }-{V}^{0})$ versus $t$ on a log-log scale. We find that as $d$ approaches closer to 45–50 nm, the replotted data of ${\rm{\log }}\,\{({V}^{\infty }-| V(t)| )/({V}^{\infty }-{V}^{0})\}$ falls on a straight line over a wider range with a slope of $-a=-\,0.4$, as indicated with a dashed line. The obtained value of $a=0.40\pm 0.05$ is nearly consistent with the theory for the DP universality class in 2D^42^, which predicts that the fraction of active (colliding) particles exhibits a power-law time dependence with an exponent $a\approx 0.45$, rather than with the theory for the *conserved* DP universality class in 2D, which predicts $a\approx 0.5$^51^.

**Figure 2:**
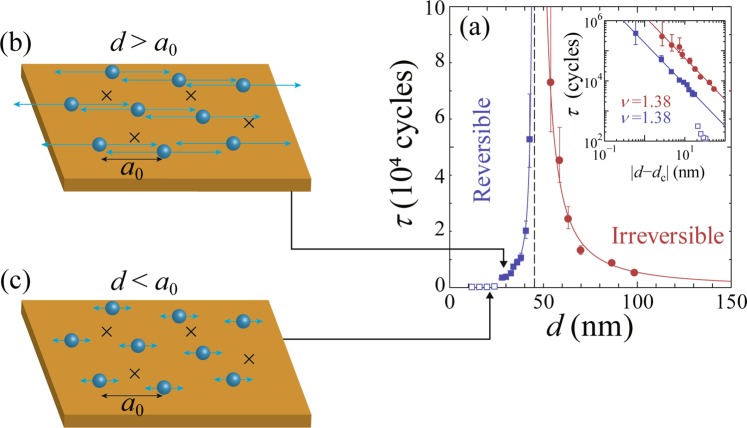
The diverging relaxation time $\tau$ at the nonequilibrium RIT. (**a**) $\tau$ plotted against $d$ for $d\,\le$ 45 nm (blue squares) and for $d\,\ge$ 50 nm (red circles), showing a power-law divergence at 45.2 $\pm$ 0.2 nm($\equiv {d}_{c}$) from both sides, as indicated with a vertical dashed line, where ${d}_{c}$ marks RIT. As $d$ is decreased to around 25 nm, which is close to the average intervortex spacing ${a}_{0}\,=$ 26 nm, $\tau (d)$ shows a downward deviation from the power-law relation, as indicated with blue open squares. Inset: ${\rm{\log }}\tau$ versus ${\rm{\log }}| d-{d}_{c}|$ plots, where symbols are the same as in the main panel. Both the red and blue lines in the main panel and inset indicate the power-law fits by $\tau \propto | d-{d}_{c}{| }^{-\nu }$ with $\nu$ = 1.38$\pm$0.08. The value of $\nu \,=$ 1.38 $\pm$ 0.08 is, within error bars, in agreement with the theoretical one $\nu =1.295\pm 0.006$ expected for the absorbing transition in the DP universality class in 2D^42^. (**b**,**c**) Schematic illustration of the periodically driven vortices over random pinning centers (crosses) with shearing amplitudes $d$ (light blue arrows) (**b**) larger than and (**c**) smaller than ${a}_{0}$.

The full lines in Fig. 1(a,b) represent the fits of $| V(t)| /{V}^{\infty }$ to Eq. (1) and the horizontal dashed lines indicate the steady-state value of $| V(t)| /{V}^{\infty }$=1. In Fig. 2(a), the values of $\tau$ thus obtained are plotted as a function of $d$ with red circles and blue squares for $d\,\ge$ 50 nm and $d\,\le$ 45 nm, respectively. They show a power-law divergence at 45.2 $\pm$ 0.2 nm ($\equiv {d}_{c}$) from both sides, as marked with a vertical dashed line. As far as we know, this is the first observation of the critical divergence of $\tau$ in the reversible state ($d < {d}_{c}$) for the strip-shaped sample with random local shear. In our previous study^18,35,36^ we measured noise spectra ${S}_{V}(f)$ in the steady state, in addition to the transient voltage $V(t)$, and estimated ${d}_{c}$ based on both measurements, whereas in this work we are unable to acquire reliable data of ${S}_{V}(f)$ due to unspecified reasons. It could be attributed to the fact that we have used the ac drive with much higher $f$ and/or the sample with slightly stronger pinning than used previously. Nevertheless, here we can determine ${d}_{c}$ from $V(t)$ with much more accuracy, since we have observed the diverging $\tau (d)$ on both sides of the transition with improved resolutions.

The inset to Fig. 2(a) displays the plots of all $$\tau $$ against $$| d-{d}_{c}| $$ on a double logarithmic scale, i.e., $${\rm{\log }}\tau $$ versus $${\rm{\log }}| d-{d}_{c}| $$, where symbols are the same as in the main panel. Both the red and blue lines in the main panel and inset indicate the power-law fits by $$\tau \propto | d-{d}_{c}{| }^{-\nu }$$ with $$\nu $$ = 1.38 $$\pm $$ 0.08. The error bars of the exponent ($$\pm $$0.08) are even smaller than those ($$\pm $$0.3^18^ and $$\pm $$0.15^36^) obtained previously. The value of $$\nu \,=$$ 1.38 $$\pm $$ 0.08 is, within error bars, in agreement with the theoretical one $$\nu =1.295\pm 0.006$$ expected for the absorbing phase transition in the DP universality class in 2D^42^. The agreement with the experiment is slightly worse for the *conserved* DP universality class in 2D^51^, which predicts a further smaller exponent $$\nu =1.225\pm 0.029$$. The observed trend in favor of DP instead of conserved DP is consistent with the experimental results of the exponent $$a$$ mentioned above. However, it is not evident whether the present vortex system in the strip sample is the one that should be described by DP or conserved DP. Here, we only point out that in the strip sample the entry and exit of vortices occur separately under the driving current, resulting in the vortex number fluctuations in time, while the average number remains constant. This is in contrast to the case of the CD sample, in which the vortices circulate around the CD center without crossing the sample edge and the vortex number is conserved at any time.

The value of the critical exponent $\nu \,=$ 1.38 $\pm$ 0.08 obtained here is clearly larger than the theoretical values of $\nu =1.105\pm 0.005$ for DP in 3D^42^ and $\nu =1.081\pm 0.027$ for conserved DP in 3D^51^, consistent with the notion that the vortex system is considered to be the 2D particles system, as treated by 2D simulations^1,11,15,16,44^. This is also supported by the experimental fact that the thickness (0.33 $\mu$m) of the film is comparable to or smaller than the magnetic penetration depth ($\sim$0.5 $\mu$m) well below ${T}_{c}$ and the possible bending distortions of vortex lines that may cause the deviation from the 2D particles picture can be ignored^25^. The value of $\nu$ = 1.38 $\pm$ 0.08 is close to $\nu$ = 1.33 $\pm$ 0.02 reported in 2D shear simulations^14^. It is also consistent, within errors, with $\nu$ = 1.3 $\pm$ 0.3 and 1.35 $\pm$ 0.15 obtained previously from the vortex experiments in CD (based on data on both sides of ${d}_{c}$)^18^ and in the strip samples (based on data on the irreversible side of ${d}_{c}$)^35,36^, respectively.

It is also found from Fig. 2(a) that as $d$ is decreased to around 25 nm in the reversible state, $\tau (d)$ shows a downward deviation from the power-law relation and takes very small values at $d\,\lesssim$ 25 nm, as indicated with blue open squares. Since 25 nm is nearly equal to the average intervortex spacing ${a}_{0}\,=$ 26 nm at 3.5 T, we interpret this behavior as reflecting the suppression of the vortex-vortex collisions at $d\lesssim {a}_{0}$. The diagrams of Fig. 2(b,c) schematically illustrate the periodically driven vortices over a random pinning potential with shearing amplitudes $d$ larger than and smaller than ${a}_{0}$, respectively. Here, the direction and amplitude of the ac motion are shown with light blue arrows, and the random pinning centers causing the local shear are schematically illustrated with crosses. It is reasonable to expect that as $d$ is decreased below ${a}_{0}$, the collisions between the vortices are less frequent and hence the random-organization process becomes less effective, resulting in the significant reduction of the relaxation $\tau$.

Meanwhile, Fig. 2(a) also shows that as $d$ is increased above ${d}_{c}(=45.2\ {\rm{nm}}\approx 2{a}_{0})$, the reversible flow transforms into the irreversible flow. At $d={d}_{c}$, the threshold vortex (particle) number, as defined as ${n}_{c}\equiv {d}_{c}/{a}_{0}(\approx 1.7)$, is about 2. In principle, this value depends on the pinning potential that causes the local shear and therefore sample dependent, while experimentally it is difficult to characterize and control the pinning properties in amorphous films. If we used a sample with a smaller pinning strength and/or pinning density, the shearing effect would be weaker and we would observe larger ${n}_{c}$. In our previous work, we have observed slightly larger values of ${n}_{c} \sim$4^35^ and $\sim$15^36^ for the strip-shaped $a$-Mo${}_{x}$Ge${}_{1-x}$ films. For example, the average pinning strength inferred from ${J}_{d}(B)$ for the latter film with ${n}_{c} \sim$ 15 is only slightly (by approximately 6$\%$) lower than that in the present film. This result suggests that ${n}_{c}$ may be more sensitive to the pinning strength than ${J}_{d}$. It may be also interesting to note that the similar threshold value of ${n}_{c}\,=$ 1–10 was reported in the colloidal suspensions^14^, although their particle density is much lower than in the vortex system and dimensionality is different.

As mentioned above, in this work we have controlled the ac displacement by changing ${V}^{\infty }$ at fixed $f$, while in our previous work^18,35,36^ $d$ was varied by changing $f$ at constant ${V}^{\infty }$. Nevertheless, we obtain nearly the same power-law exponents $\nu$ from both experiments. This result indicates that the critical behavior associated with the transient dynamics near RIT is independent of the details of the parameters used to change $d$, that is, independent of whether ${V}^{\infty }$ (the velocity) or $f$ (the driving time) is varied in the ${V}^{\infty }$ and $f$ ranges studied.

**Figure 3:**
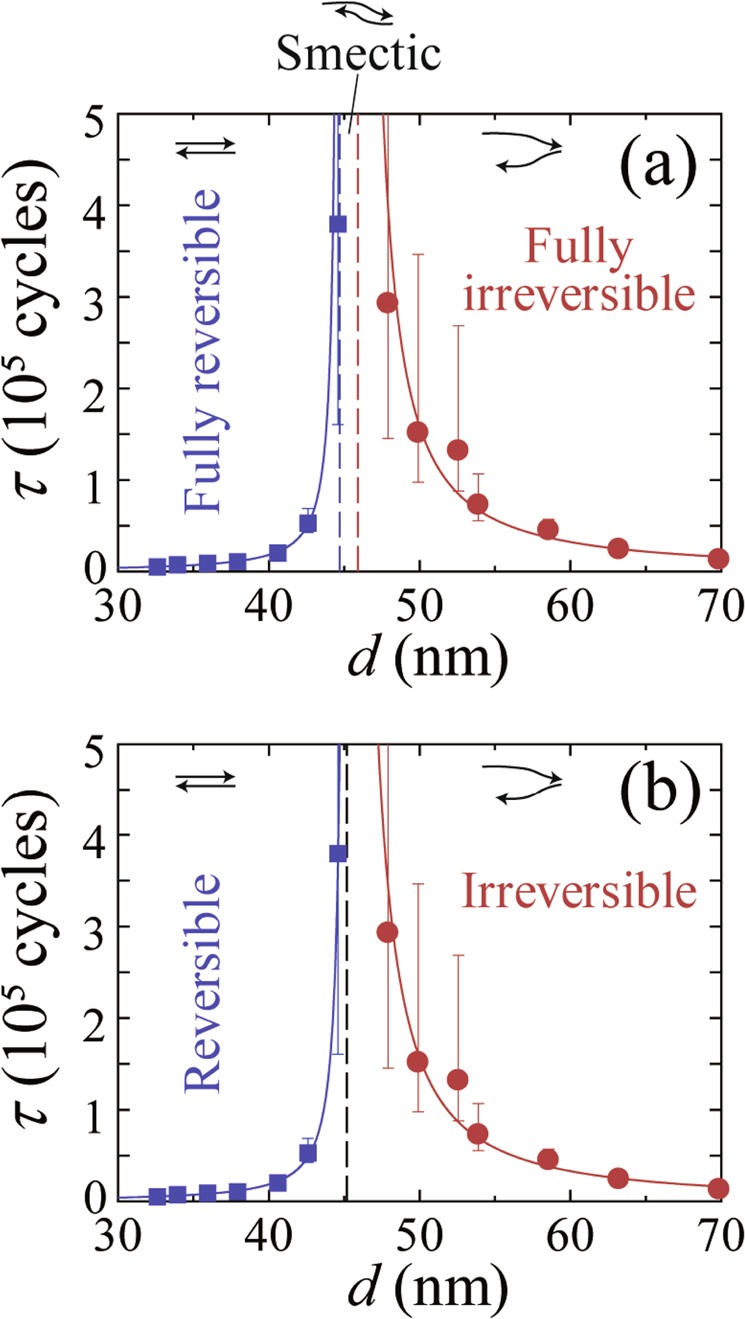
The possible smectic-flow regime intervening between reversible and irreversible flow. (**a**) $\tau$ versus $d$ around the transition region. Both the blue and red lines indicate the power-law fits with ${d}_{c1}\,=$ 44.7 and ${d}_{c2}\,=$ 45.9 nm, respectively, where we fixed a value of the critical exponent to be $\nu$=1.295 expected for the DP universality class in 2D^42^. The vertical blue and red dashed lines mark the position of ${d}_{c1}$ and ${d}_{c2}$, respectively. (**b**) The same fits as in the main panel of Fig. 2(a) are shown with blue and red lines, where ${d}_{c}\,=$ 45.2 nm and $\nu \,=$1.38 $\pm$ 0.08 are obtained by the fitting. A vertical dashed line marks the position of ${d}_{c}\,=$ 45.2 nm, which separates reversible and irreversible flow. Although we cannot conclude definitely which of the fits in (**a**) or (**b**) is better, assuming the theoretical value of $\nu$=1.295^42^ and using the simulation results^44^, there may be a narrow smectic regime (${d}_{c1} < d < {d}_{c2}$) between fully reversible flow ($d < {d}_{c1}$) and fully irreversible flow ($d > {d}_{c2}$). Insets: Schematic illustration of the vortex motion within a shear cycle in the steady state for different flow regimes.

Summarizing what has been presented above, we observe the relaxation time $\tau$ that diverges at ${d}_{c}\,=$ 45.2 nm from both sides of the transition and obtain the accurate value of the critical exponent $\nu =1.38$, which is, within error bars, in agreement with $\nu =1.295$ expected for the absorbing transition in the 2D DP universality class^42^. More strictly, however, the value of $\nu$ = 1.38 looks slightly larger than $\nu =1.295$. Here, it is noted that $\nu$ obtained from the power-law fit, $\tau \propto | d-{d}_{c}{| }^{-\nu }$, correlates with ${d}_{c}$. Thus, let us heuristically estimate the optimal ${d}_{c}$ from the power-law fit, *assuming* that the critical exponent is fixed to be $\nu =1.295$ expected for DP in 2D. Our results show that we cannot fit the data well using a single value of ${d}_{c}$, but we can do using slightly different two values, ${d}_{c1}\,=$ 44.7 and ${d}_{c2}\,=$ 45.9 nm, instead of ${d}_{c}\,=$ 45.2 nm. The results of the fits are shown in Fig. 3(a), where the vertical blue and red dashed lines mark the location of ${d}_{c1}$ and ${d}_{c2}$, respectively, and both the blue and red full lines represent the power-law fits with ${d}_{c1}\,=$ 44.7 and ${d}_{c2}\,=$ 45.9 nm, respectively, and a common value of $\nu$ = 1.295. For comparison, the same fits as in the main panel of Fig. 2 (a) are shown in Fig. 3 (b), where the transition region around $d \sim {d}_{c}$ is enlarged and shown. Since the gap between ${d}_{c1}$ and ${d}_{c2}$ is very small, which corresponds to $({d}_{c2}-{d}_{c1})/{d}_{c1}=(45.9-44.7)/44.7\,=$ 0.03, we cannot conclude definitely which of the two fits shown in Fig. 3 (a,b) is better.

According to the recent simulation^44^ for a periodically sheared overdamped particles over quenched disorder, such as the vortex system studied in this work, there are two transition points for RIT, which are characterized by two values of ${d}_{c}$ depending on whether one approaches the transition from the reversible side or the irreversible side. The values of ${d}_{c}$ derived by the power-law fits in the reversible and irreversible states are ${d}_{c1}=$24.6 and ${d}_{c2}=$25.8, respectively, where $\nu$=1.33 and 1.31. The relative gap size in the simulation is $({d}_{c2}-{d}_{c1})/{d}_{c1}=(25.8-24.6)/24.6\,=$ 0.05, which is similar to the experimental one $({d}_{c2}-{d}_{c1})/{d}_{c1}\,=$ 0.03 obtained here, although this value becomes slightly smaller using $\nu \,=$ 1.33 or 1.31 instead of $\nu =1.295$. The simulation^44^ has shown that the first transition at ${d}_{c1}$ is from fully reversible flow to a smectic state where the flow is reversible transverse to the drive but irreversible in the direction of the drive. The second transition at ${d}_{c2}$ is from this smectic flow to fully irreversible flow. They are schematically illustrated in the insets to Fig. 3(a,b). While it is of great interest to verify this prediction by more direct experiments, it is difficult to measure the relaxation transverse to the drive in the actual samples. This is because the Hall component of the voltage is much smaller than the longitudinal voltage and the signal to noise ratio is significantly small due to the longitudinal component originating from the slight misalignment of the Hall-voltage probes, added to the transverse voltage.

We expect that the present work will stimulate further research on RIT, e.g., in the presence of isotropic shear where smectic flow is considered to be absent, and on nonequilibrium phase transitions in various many-particle systems, including colloidal particles^52,53^ or dense, jammed systems^28,54–59^.

## Conclusions

We study the general phenomenon of RIT by measuring the transient dynamics of vortices driven periodically in the rectangular $a$-Mo${}_{x}$Ge${}_{1-x}$ film with random pinning that causes random local shear. In the vicinity of RIT, the fraction of active (colliding) vortices estimated from ${V}^{\infty }-| V(t)|$ exhibits a power-law time dependence with an exponent of $a\approx 0.4$, which is close to $a\approx 0.45$ expected for the absorbing phase transition in the DP universality class in 2D^42^. The relaxation time $\tau (d)$ for the system to reach either the reversible or irreversible state shows a power-law divergence at the threshold displacement. The critical exponent $\nu =1.38\pm 0.08$ is determined with higher accuracy than in previous experiments^18,35,36^ and is, within error bars, in agreement with the value $\nu$ = 1.295 $\pm$ 0.006 predicted for the absorbing transition in the DP universality class in 2D^42^ again. These values of $\nu$ are similar to ${\nu }_{d}\,\approx$ 1.4 for the nonequilibrium depinning transition obtained in the same vortex system from the data on the moving (fluctuating) side of the transition^18,19^, consistent with the prediction that both transitions may fall into the same universality class as the absorbing transition in 2D DP^5^.

As $d$ is decreased down to the mean intervortex spacing ${a}_{0}$ in the reversible regime, $\tau (d)$ deviates downward from the power-law relation, reflecting the suppression of vortex-vortex collisions and of random organization at $d\le {a}_{0}$. Assuming the theoretical value of $\nu$ = 1.295 for the 2D DP class and using simulation results with anisotropic periodic shear^44^, we suggest the possibility of the narrow smectic-flow regime intervening between the fully reversible and irreversible flow states.
